# Authorship growth in contemporary medical literature

**DOI:** 10.1177/2050312120915399

**Published:** 2020-03-30

**Authors:** Julie Y An, Rachel J Marchalik, Rachael L Sherrer, Joseph A Baiocco, Soroush Rais-Bahrami

**Affiliations:** 1 Medical Research Scholars Program, National Institutes of Health, Bethesda, MD, USA; 2 Department of Radiology, University of California–San Diego, San Diego, CA, USA; 3 Department of Medicine, MedStar Washington Hospital Center, Washington DC, USA; 4 Department of Urology, University of Wisconsin–Madison, Madison, WI, USA; 5 Department of Urology, Icahn School of Medicine at Mount Sinai, New York, NY, USA; 6 Department of Urology, The University of Alabama at Birmingham, Birmingham, AL, USA; 7 Department of Radiology, The University of Alabama at Birmingham, Birmingham, AL, USA

**Keywords:** Citations, impact factor, academic medicine, medical specialties, medical publishing

## Abstract

**Objectives:**

The aims of this study were to investigate authorship trends among publications in high-impact, peer-reviewed specialty journals published within the last decade and to assess how publication practices differ among medical specialties.

**Methods:**

The National Institutes of Health’s Portfolio Analysis platform, iCite, was queried for PubMed-indexed case reports, review articles, and original research articles published between 2005 and 2017 in 69 high-impact, clinical journals encompassing 23 medical specialties. Overall, 121,397 peer-reviewed publications were evaluated—of which, 45.1% were original research, 28.7% were review articles, and 26.3% were case reports. Multivariable regression was used to evaluate the magnitude of association of publication year on the number of authors per article by specialty and article type.

**Results:**

Original research articles have the greatest increase in authorship (0.23 more authors per article per year), as compared with review articles (0.18 authors per article per year) and case reports (0.01 authors per article per year). Twenty-two of the 23 specialties evaluated had increase in authorship in high-impact specialty journals. Specialty growth rates ranged from 0.42 authors/year (Neurology), Psychiatry (0.35 authors/year), General Surgery (0.29 authors/year), Urology (0.27 authors/year), and Pathology (0.27 authors/year). Specialties with a greater percentage of graduates entering academics had more authors per article; surgical specialties and length of residency were not found to be predictive factors.

**Conclusion:**

There has been substantial growth in the authorship bylines of contemporary medical literature, much of which cannot be explained by increased complexity or collaboration alone.

## Introduction

Collaboration within medical research accelerates innovation and promotes complex scientific endeavors. Technological advances facilitating communication and the ability to record and share data have simplified the collaborative process, revolutionizing contemporary research practices. Not surprisingly, research has grown in both scale and rigor. Today, multi-disciplinary and multi-institutional collaborations are the norm and are often considered necessary for maintaining and achieving the highest clinical research standards.

This shift has been accompanied by a growth in the number of authors per scientific manuscript. Single and few-authored papers, common in decades prior, are exceedingly rare today. This is especially true in publications with original data. Curiously, these trends appear to be pervasive in nearly all-academic fields. *The Economist* recently observed increasing authorship trends in various academic disciplines, including Arts & Humanities, Economics, Engineering, Chemistry, Medicine, and Physics & Astronomy. Notably, medicine had the second greatest rate of authorship growth, behind publications in Physics & Astronomy.^1^ Increasing authorship has been independently observed in Pediatric Surgery,^2^ Radiology,^3^ Radiation Oncology,^4^ Neurology,^5^ Urology,^6^ Sports Medicine,^7^ Orthopedics,^8^ Psychiatry,^9^ and Obstetrics and Gynecology (OB/GYN)^10^ literature, but without systematic comparisons.

It has been proposed that the increased authorship stems from the need for greater manpower to conduct high-quality research endeavors.^11^ Others attribute increased authorship to widespread authorship inflation driven by growing academic pressures, decreased funding resources, and variable measures for academic promotion across fields and institutions.^10,12,13^ The exact driving forces behind this trend are uncertain and likely multifactorial.

Traditionally, academic credit is awarded to individuals who make “sufficient” contributions in the form of authorship. The International Committee of Medical Journal Editors (ICMJE)^14^ defined authorship as fulfilling the following four criteria: (1) substantial contributions to the conception or analysis of data, (2) drafting the work or revising it critically for important intellectual content, (3) final approval of submitted manuscript, and (4) agreement to be accountable for all aspects of the work. Contributors who do not fulfill these four criteria should be credited through an acknowledgment. However, in situations where many collaborators have contributed in varying degrees and forms, it can be difficult to discern whose contributions justify authorship and to what capacity. In this study, we aim to describe authorship trends within contemporary medical literature and to assess how these patterns compare across different medical specialties. Ultimately, the goal of this research is to elucidate the driving forces behind this change and promote the implementation of standards that guide authors to appropriately assign authorship.

## Methods

### Specialty, journals, and article information

The Association of American Medical Colleges^15^ (AAMC, Washington, DC) Careers in Medicine^®^ tool and the Report on Residents^®^ were used to identify 23 medical specialties in which training is entered directly by graduating senior medical students.^16^ Information including years of residency training, percentage of recent graduates with academic faculty positions, and surgical versus non-surgical specialty were collected. The 2016 Journal Citation Reports^®17^ (Thomson Reuter, New York) was used to identify three of the highest impact-factored, clinically orientated, specialty-specific publications for each of the 23 specialties. The National Institutes of Health’s (NIH) National Library of Medicine PubMed database was used to obtain PubMed IDs (PMID) of case reports, review articles, and original research published between 1 January 2005 and 31 December 2017 in the 69 selected journals included in this study. We excluded all articles listed under addresses, biographies, comment, dataset, editorials, abstracts, guidelines, personal narratives, erratum, and video–audio media.

The NIH Office of Portfolio Analysis’s web-based next-generation portfolio analysis platform, iCite, was queried for bibliometric data including article title, author list, year of publication, article type, journal in which it was published, and relative citation ratio (RCR, a field-normalized metric which shows the scientific influence of each article relative to the average NIH-funded article in the same discipline published in the same year) for each article selected for analysis.^18^ Permission and assistance with use of the iCite program were obtained from the program developers in Bethesda, Maryland, in 2017 when this study was first conceptualized. Article type was categorized strictly based on how they were recorded in the PubMed database to maintain consistency between classification between journals. This observational study did not involve human subjects and was therefore exempt from institutional ethics review.

### Statistical analysis

Statistical analyses were performed on Stata/SE 14 (StataCorp LLC, College Station, Texas), with two-sided p-values < 0.05 predetermined as the threshold for statistical significance. Article citation characteristics were described by article type, by specialty, and for all articles. One-way analysis of variance (ANOVA) compared authors per article, number of total citations, citations per year, and RCR by article type. Multivariable linear regression analysis of non-collinear variables (article year, specialty, article type) as predictors of authors per article was performed and used to calculate predicted values. Stratified regression models were repeated independently within each article type (with specialty as co-variable) and within each specialty (with article type as co-variable). Adjusted coefficients for publication year on authors per article were reported; analyses were performed for all articles, by article type, and by specialty. Predicted values from the three article-type stratified regression models were used to produce a tabular and several graphical illustrations. Specialty characteristics including surgical versus non-surgical specialty type, minimum years of residency training, and percent of graduates with current academic positions were evaluated using multivariable regression as potential predictors of mean authors per specialty article. Ninety-five percent confidence interval (CI) and standard errors of coefficients were calculated using the bootstrap sampling with 50 replications.

## Results

### Summary of specialty, journal, and articles evaluated

**Figure 1.**
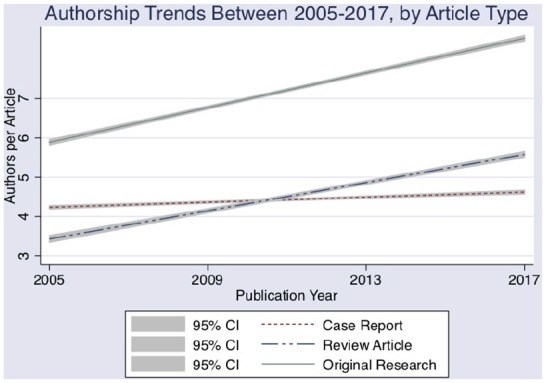
Authorship trends in medical peer-reviewed publications from 2005 to 2017 by article type.

Overall, 121,397 peer-reviewed publications were evaluated—of which, 45.1% were original research, 28.7% review articles, and 26.3% case reports (Table 1). Original research had significantly more authors per article (7.26 authors, adjusted mean), than review articles (4.63 authors, adjusted mean) and case reports (4.46 authors, adjusted mean) (ANOVA: p < 0.001). Average RCR, metric for article impact, was similarly greatest in review articles (RCR: 2.98), followed by original research (RCR: 2.44) and case report (RCR: 0.54) (ANOVA: p < 0.001) (Table 1). Each consecutive year was associated with an increase of 0.16 authors per publication for all articles and specialties combined (p < 0.001) (Table 2). Original research articles were associated with the greatest authorship growth (growth of 0.23 authors/year, an increase from 5.87 to 8.51 authors between 2005 and 2017), as compared with review articles (0.18 authors/year, 3.53 to 5.69 authors) and case reports (0.01 authors/year, 4.26 to 4.49 authors) (p < 0.001 for all) (Figure 1, Table 2). Case reports initially had more authors than review articles, but this reversed over time—with review articles later having more authors than case reports by about 2010 (Table 3, Figure 1). This trend was also seen in the majority of evaluated specialties (Figure 3).

**Table 1.** Summary of articles, citations, and RCR by specialty evaluated.

	Citations	Citations per year	RCR
	p < 0.001	p < 0.001	p < 0.001
By article type			
Case report (n = 31,877)	3 (1–8)	0.4 (0.1–1.0)	0.3 (0.1–0.6)
Review article (n = 34,799)	15 (4–40)	2.7 (1.0–6.2)	1.6 (0.6–3.5)
Original research (n = 54,721)	18 (7–38)	2.7 (1.3–5.2)	1.5 (0.8–2.9)
By specialty			
Anesthesiology (n = 5515)	14 (5–34)	2 (0.7–4.7)	1.2 (0.4–2.7)
*Anaesthesia*: n = 1658 (30.1%)			
*Anesthesiology*: n = 1856 (33.7%)			
*Br J Anaesth*: n = 2001 (36.3%)			
Dermatology (n = 5683)	7 (2–21)	1.3 (0.4–3.6)	0.8 (0.3–1.9)
*J Am Acad Dermatol*: n = 3858 (67.9%)			
*J Invest Dermatol*: n = 1014 (17.8%)			
*JAMA Dermatol*: n = 811 (14.3%)			
Emergency Medicine (n = 3129)	7 (1–21)	1.3 (0.2–3.0)	0.8 (0.2–1.8)
*Acad Emerg Med*: n = 1100 (35.2%)			
*Ann Emerg Med*: n = 1656 (52.9%)			
*Scand J Trauma Resusc Emerg Med*: n = 373 (11.9%)			
Family Medicine (n = 1234)	11 (3–28)	1.7 (0.7–3.4)	1.0 (0.4–1.9)
*Ann Fam Med*: n = 318 (25.8%)			
*Br J Gen Pract*: n = 462 (37.4%)			
*J Am Board Fam Med*: n = 454 (36.8%)			
General Surgery (n = 3514)	22 (7–50)	4.6 (2.0–8.5)	2.6 (1.2–4.7)
*Ann Surg*: n = 1379 (39.2%)			
*Br J Surg*: n = 1683 (47.9%)			
*JAMA Surg*: n = 452 (12.9%)			
Internal Medicine (n = 3524)	28 (7–78)	5.5 (1.7–12.5)	2.5 (0.8–5.5)
*Ann Intern Med*: n = 2029 (57.6%)			
*J Intern Med*: n = 836 (23.7%)			
*JAMA Intern Med*: n = 659 (18.7%)			
Interventional Radiology (n = 4247)	4 (1–11)	0.7 (0.2–1.8)	0.5 (0.1–1.1)
*Cardiovasc Intervent Radiol*: n = 1627 (38.3%)			
*J Vasc Interv Radiol*: n = 2052 (48.3%)			
*Semin Intervent Radiol*: n = 568 (13.4%)			
Neurology (n = 2594)	48 (15–109)	8 (3.5–15.5)	3.5 (1.6–6.6)
*Brain*: n = 899 (34.7%)			
*JAMA Neurol*: n = 529 (20.4%)			
*Lancet Neurol*: n = 1166 (44.9%)			
Neurosurgery (n = 7569)	8 (2–21)	1.3 (0.5–3.0)	1.0 (0.4–2.0)
*J Neurosurg*: n = 2121 (28.0%)			
*Neurosurgery*: n = 2976 (39.3%)			
*World Neurosurg*: n = 2472 (32.7%)			
Obstetrics & Gynecology (n = 6743)	15 (5–35)	2.1 (0.8–4.7)	1.2 (0.5–2.6)
*Am J Obstet Gynecol*: n = 2489 (36.9%)			
*Hum Reprod*: n = 1366 (20.3%)			
*Obstet Gynecol*: n = 2888 (42.8%)			
Ophthalmology (n = 5267)	14 (4–34)	2.3 (0.8–5.0)	1.5 (0.6–3.2)
*Am J Ophthalmol*: n = 1869 (35.5%)			
*JAMA Ophthalmol*: n = 814 (15.5%)			
*Ophthalmology*: n = 2584 (49.1%)			
Orthopedic Surgery (n = 5841)	15 (5–34)	2.2 (1.0–4.7)	1.6 (0.7–3.2)
*Clin Orthop Relat Res*: n = 2496 (42.7%)			
*J Am Acad Orthop Surg*: n = 996 (17.1%)			
*J Bone Joint Surg Am*: n = 2349 (40.2%)			
Otolaryngology (n = 3562)	5 (1–13)	0.8 (0.2–1.9)	0.6 (0.2–1.4)
*Clin Otolaryngol*: n = 567 (15.9%)			
*JAMA Otolaryngol Head Neck Surg*: n = 500 (14.0%)			
*Otolaryngol Head Neck Surg*: n = 2495 (70.0%)			
Pathology (n = 2073)	24 (10–49)	3.2 (10–49)	1.5 (0.8–2.7)
*Am J Surg Pathol*: n = 959 (46.3%)			
*J Pathol*: n = 505 (24.4%)			
*Mod Pathol*: n = 609 (29.4%)			
Pediatrics (n = 7977)	16 (5–38)	2.7 (1.0–5.5)	1.4 (0.6–3.0)
*J Pediatr*: n = 2522 (31.6%)			
*JAMA Pediatr*: n = 404 (5.1%)			
*Pediatrics*: n = 5051 (63.3%)			
Physical Medicine & Rehabilitation (n = 3981)	9 (3–22)	1.6 (0.6–3.3)	1.1 (0.5–2.2)
*Am J Phys Med Rehabil*: n = 1256 (31.5%)			
*Arch Phys Med Rehabil*: n = 2234 (56.1%)			
*Eur J Phys Rehabil Med*: n = 491 (12.3%)			
Plastic Surgery (n = 6174)	6 (2–15)	0.8 (0.2–15)	0.7 (0.2–1.8)
*J Plast Reconstr Aesthet Surg*: n = 2521 (40.8%)			
*JAMA Facial Plast Surg*: n = 137 (2.2%)			
*Plast Reconstr Surg*: n = 3516 (56.9%)			
Psychiatry (n = 2929)	38 (14–85)	5.3 (2.2–10.5)	2.8 (1.3–5.2)
*Am J Psychiatry*: n = 1776 (60.6%)			
*Br J Psychiatry*: n = 849 (29.0%)			
*JAMA Psychiatry*: n = 304 (10.4%)			
Radiation Oncology (n = 4580)	19 (8–40)	3.0 (1.5–5.4)	1.4 (0.7–2.5)
*Int J Radiat Oncol Biol Phys*: n = 2709 (59.1%)			
*Radiother Oncol*: n = 1443 (31.5%)			
*Semin Radiat Oncol*: n = 428 (9.3%)			
Radiology (n = 5809)	13 (4–32)	1.8 (0.7–4.1)	1.1 (0.5–2.4)
*AJR Am J Roentgenol*: n = 3166 (54.5%)			
*J Am Coll Radiol*: n = 701 (12.1%)			
*Radiology*: n = 1942 (33.4%)			
Thoracic Surgery (n = 12,848)	5 (1–16)	0.8 (0.2–2.5)	0.5 (0.1–1.3)
*Ann Thorac Surg*: n = 7037 (54.8%)			
*Eur J Cardiothorac Surg*: n = 2684 (20.9%)			
*J Thorac Cardiovasc Surg*: n = 3127 (24.3%)			
Urology (n = 8932)	15 (4–36)	2.1 (0.7–5.0)	1.1 (0.4–2.5)
*Eur Urol*: n = 2056 (23.0%)			
*J Urol*: n = 3317 (37.1%)			
*Urology*: n = 3559 (39.8%)			
Vascular Surgery (n = 7672)	7 (2–19)	1.1 (0.4–3.0)	0.7 (0.3–1.6)
*Ann Vasc Surg*: n = 2345 (30.6%)			
*Eur J Vasc Endovasc Surg*: n = 1654 (21.6%)			
*Vasc Surg*: n = 1654 (21.6%)			
Overall			
Total (n = 121,397)	11 (3–30)	1.8 (0.5–4.3)	1.1 (0.4–2.5)

RCR: relative citation ratio; ANOVA: analysis of variance.

Citation count, citations per year, and RCR were compared by article type and by specialty using the one-way ANOVA test. Journals names written in PubMed MedAbbr format. All values presented as median (interquartile range) unless otherwise specified.

**Table 2.** Multivariable linear regression analysis of non-collinear variables (specialty, article type, and year) as predictors of authors per article.

	Coefficient	p-value	95% CI
By article type			
Case report (n = 31,877)	(Reference)		
Review article (n = 34,799)	0.04	0.119	[–0.01, 0.1]
Original research (n = 54,721)	2.67	<0.001	[2.62, 2.72]
By specialty			
Orthopedic Surgery (n = 5841)	(Reference)		
Otolaryngology (n = 3562)	0.02	0.669	[–0.09, 0.13]
Physical Medicine & Rehabilitation (n = 3981)	0.11	0.074	[–0.01, 0.22]
Plastic Surgery (n = 6174)	0.12	0.003	[0.04, 0.19]
Family Medicine (n = 1234)	0.13	0.110	[–0.03, 0.29]
Anesthesiology (n = 5515)	0.45	<0.001	[0.35, 0.55]
Emergency Medicine (n = 3129)	0.49	<0.001	[0.35, 0.62]
Obstetrics & Gynecology (n = 6743)	0.84	<0.001	[0.75, 0.93]
Vascular Surgery (n = 7672)	0.90	<0.001	[0.81, 0.99]
Ophthalmology (n = 5267)	1.10	<0.001	[0.98, 1.22]
Interventional Radiology (n = 4247)	1.25	<0.001	[1.12, 1.39]
Pediatrics (n = 7977)	1.25	<0.001	[1.15, 1.36]
Dermatology (n = 5683)	1.28	<0.001	[1.16, 1.41]
Radiology (n = 5809)	1.30	<0.001	[1.19, 1.41]
Neurosurgery (n = 7569)	1.41	<0.001	[1.32, 1.49]
Thoracic Surgery (n = 12,848)	1.64	<0.001	[1.56, 1.71]
General Surgery (n = 3514)	1.71	<0.001	[1.56, 1.85]
Urology (n = 8932)	1.71	<0.001	[1.6, 1.82]
Internal Medicine (n = 3524)	1.83	<0.001	[1.64, 2.02]
Psychiatry (n = 2929)	2.19	<0.001	[1.96, 2.42]
Pathology (n = 2073)	2.36	<0.001	[2.16, 2.57]
Radiation Oncology (n = 4580)	2.97	<0.001	[2.83, 3.11]
Neurology (n = 2594)	4.16	<0.001	[3.80, 4.52]
By Year			
Year	0.16	<0.001	[0.15, 0.16]

CI: confidence interval.

Variables are ordered from smallest to largest coefficient values.

**Table 3.** Authors per article by year, predicted from article-type stratified regression models.

	Case report		Review article		Original research	
	Mean	SE	Mean	SE	Mean	SE
Authors per article, by year						
2005	4.26	0.01	3.53	0.01	5.87	0.01
2006	4.27	0.01	3.67	0.01	6.03	0.01
2007	4.29	0.01	3.87	0.01	6.31	0.01
2008	4.32	0.01	4.01	0.01	6.48	0.01
2009	4.33	0.01	4.22	0.01	6.71	0.01
2010	4.38	0.01	4.39	0.01	6.93	0.02
2011	4.35	0.01	4.59	0.01	7.09	0.01
2012	4.37	0.01	4.78	0.01	7.32	0.01
2013	4.43	0.01	4.98	0.01	7.68	0.01
2014	4.45	0.01	5.16	0.01	7.91	0.01
2015	4.42	0.01	5.32	0.01	8.11	0.01
2016	4.50	0.01	5.53	0.01	8.36	0.01
2017	4.49	0.01	5.69	0.01	8.51	0.01
Authors per article, overall						
2005–2017	4.46	0.00	4.63	0.00	7.26	0.00

SE: standard error.

Predicted values were obtained from the linear regression model fitted and adjusted for authorship year, article type, and specialty.

### Authorship trends by specialty

**Figure 2.**
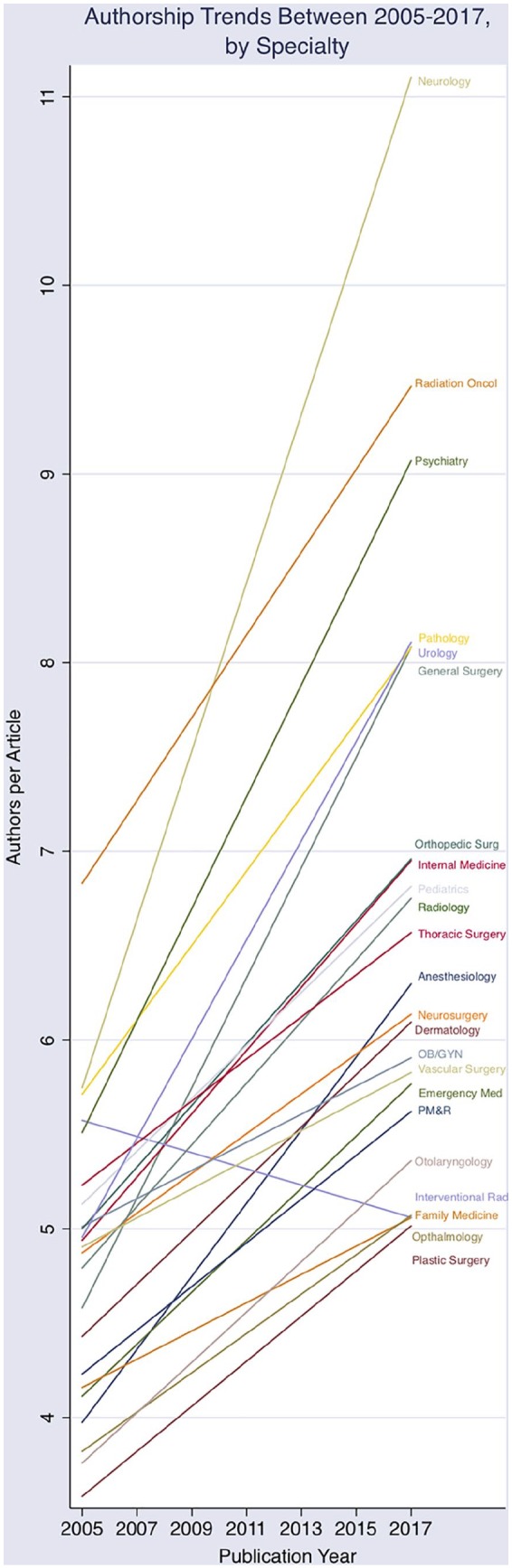
Authorship trends in medical peer-reviewed publications from 2005 to 2017 by specialty.

**Figure 3.**
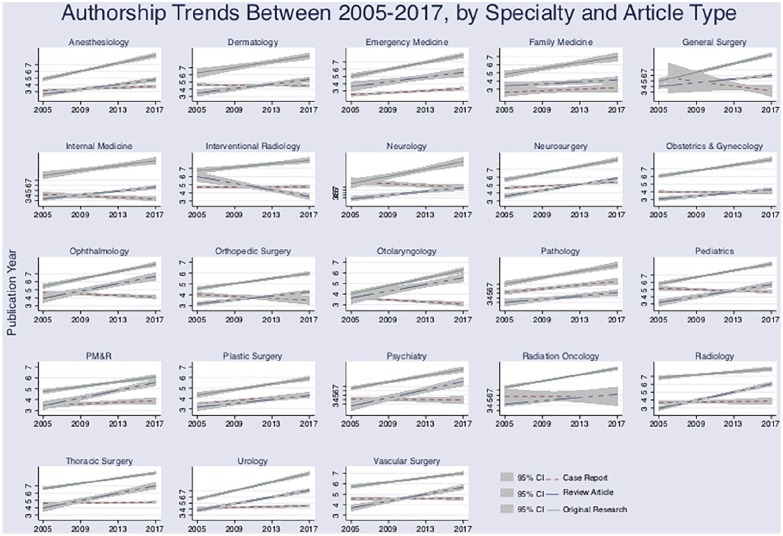
Authorship trends in medical peer-reviewed publications from 2005 to 2017 by article type within individual specialty.

Specialties with the greatest number of authors per article were as follows: Neurology, Radiation Oncology, Pathology, Psychiatry, and Internal Medicine (from greatest to less). Specialties with the fewest authors per article were as follows: Orthopedic Surgery, Otolaryngology, Physical Medicine and Rehabilitation, and Plastic Surgery. Orthopedic Surgery had the fewest adjusted authors per article, whereas Neurology had the most—4.16 more authors than Orthopedic Surgery (Table 2). The majority (22/23) of all specialties independently experienced statistically significant adjusted authorship growth for all articles (p < 0.001 for these 22 specialties). Interventional Radiology was the only specialty that did not show a statistically significant authorship growth or decline (p = 0.12). Specialties with the greatest authorship growth were Neurology (growth of 0.42 authors/year), Psychiatry (0.35 authors/year), General Surgery (0.29 authors/year), Urology (0.27 authors/year), and Pathology (0.27 authors/year). Specialties with the least authorship growth were Vascular Surgery (0.09 authors/year), Dermatology (0.10 authors/year), Orthopedic Surgery (0.10 authors/year), Plastic Surgery (0.10 authors/year), and Thoracic Surgery (0.10 authors/year). Graphical comparison of authorship growth, by specialty, is presented in Figure 2, and by article type, within each specialty, is presented in Figure 3.

### Specialty factors associated with number of authors per article

Specialties with more graduates entering academic practice were associated with more authors per article. Each percent increase in graduates entering academics was associated to 0.11 (95% CI = [0.04, 0.19]) more authors per article (p = 0.01). Neither surgical specialty (p = 0.36) nor length of residency training (p = 0.11) was found to be associated factors in the adjusted model.

## Discussion

The goal of this study was to generate data that describe authorship trends in contemporary medical literature in order to open a dialogue about authorship practices. Ambiguous recommendations, and variability in institution-based customs and politics, make this a challenging topic that few have openly discussed and defined. Appropriate authorship designation is imperative for maintaining the credibility of medical research. However, since citation counting has become the established means to determine academic prominence, it has also become a system which can be “gamed” with appropriate awareness of the standards. The original metrics of academic productivity may now have ceased to hold the same relations to the outcome measures they were designed to assess.

In our evaluation of 31,877 case reports, 34,799 review articles, and 54,721 original research articles, we identified a global increase in the number of authors per article over the past 12 years across article types and specialties. Publication year was found to be an independent predictor of higher number of authors per publication. Each consecutive year is associated with 0.16 more authors per peer-reviewed article. Although it is conceivable that increased collaboration in big-data-driven studies is responsible for this change, certain findings appear to contradict that hypothesis. For example, the number of authors should not have increased for review articles, as the complexity or collaborative efforts needed to write this type of publication have not increased over the last decade. This leads us to suspect that authorship inflation may have contributed to the observed increased authorship trend.

We were also interested to know how authorship trends compared between surgical and non-surgical specialties, which specialty characteristics impact bibliometric measures, and the nature of this impact. While all physicians first undergo similar 4-year training during medical school, training lengths and academic interests diverge dramatically during residency training. Certain specialties and specific training programs place greater emphasis on research and evaluate for participation when screening applicants.^19^ Therefore, we suspected more competitive residency specialties and those known to place more emphasis upon academic participation would have more authors per article and/or show a greater number or growth in authorship.

The only characteristic we observed to be an associated factor was percentage of graduates with academic involvement after residency. Physicians who spend greater time in academic environments are more likely to be influenced by, and participate in, interdisciplinary medical research based upon their surroundings. Interestingly, surgical specialties, or the perceived competitiveness of a specialty’s match, do not appear to be a deterministic factor of authorship quantity. The five specialties with the greatest number of authors per article (adjusted for article type and year of publication) range in competitiveness, procedural capacity, and primary care designations—Neurology, Radiation Oncology, Pathology, Psychiatry, and Internal Medicine. Both environmental influences during training and self-selection of residency applicants likely influenced specialty-publishing behaviors.

### Measuring productivity in academics

The primary way an individual researcher’s productivity, regardless of academic discipline, is quantified by way of the H-index. The H-index is an author-level metric derived from citations of his or her published works.^20^ Authors whose names appear on more papers have a greater likelihood of reaching a higher H-index with more citations, which in turn benefits them in both their academic standing and potentially their ability to secure research time and funding. The H-index is not immune to inflation by means of self-citations, so authors have a theoretical incentive to cite their previous work. Although our current study did not aim to address the issue of self-citations, we did observe a positive, independent association between increased authorship and number of citations an article received. There is inherent bias to the simple H-index where self-citations can artificially increase an author’s H-index, and hence modifications excluding self-citations have been proposed.^21^

Another strategy to increase one’s H-index is to be an author on a piece of work with a broader audience with potential for greater visibility. It was notable that, overall, and for most specialties, case reports initially had more authors than review articles, and with time, this trend reversed—with review articles now having more authors than case reports. We suspect this shift may be related to the greater awareness that review articles garner more citations (mean RCR for review articles = 2.98, as compared with that of case reports = 0.54). Though each measure of publication impact is prone to being manipulated by authors seeking increased visibility and recognition, all are largely based on the reputation of the authors in the byline and the publications in which they are published.^22^ There has also been a significant movement toward quantitative metrics including Altmetric for measure of publication prolificacy and post-publication value. This metric takes into account the impact of electronic citations in the ever-increasing era of electronic communication and social media prominence.^23^

### Exploring the “publish or perish” mentality

The professional stature of an academician is measured by the number of publications, impact factor of the journals publishing the articles, and by number of citations the work receives following publication. It is these mounting pressures to raise or maintain academic reputation that can lead to authorship inflation.^24^ Honorary or gift authorships—the inclusion of a well-respected researcher for political reasons or to bring more merit to an article—and inclusion of junior authors for purposes of building academic rapport are both known forms of authorship inflation.

In our current system of calculating an author’s H-index, all co-authors are rewarded the same credit regardless of the number and order in which these authors’ names appear. Authorship inflation and self-citation undoubtedly allow researchers to gain greater visibility within their field, and individuals may feel pressured to partake in this practice because those not participating are at a relative disadvantage compared to those who do. There is little incentive to be conservative with authorship when the alternative is more likely to yield greater benefit in visibility and subsequent citation potential.

The ICMJE released guidelines on required authorship contributions, the most well-established set of guidelines used across most medical journals, was published in an effort to standardize practice and guide authors faced with authorship disputes. Despite the ICMJE’s attempt to regulate authorship assignment, there is little evidence to show ICMJE guidelines have altered any publication practices.^25^ Not all medical journals require compliance with ICMJE Uniform Recommendations, and authors submitting to journals requiring compliance may not fully understand the specified recommendations.^25,26^ For example, a survey of over 300 healthcare professionals found that 33% of respondents believe “general supervision of a research lab” was sufficient to merit authorship.^27^ Interestingly, there was no difference in the level of understanding of appropriate authorship criteria when respondents were aware or unaware of the guidelines. In a separate survey of 119 American medical, veterinary, and dental students enrolled in research fellowships at either the NIH or a sponsored academic medical center, 66% of respondents reported never receiving formal training in authorship guidelines.^28^

Although there is a need for greater ethical publishing practices, simple promotion of good practices is not enough and unlikely to result in significant changes on its own. Dedicated training, formal discussion with research mentors, and regarding authorship may be valuable to both seasoned researchers and trainees. In order to develop a generation of ethical clinician scientists, current leaders must lead by example and realize their actions have a trickle-down effect on those they train. Physicians and scientists who understand are more likely to attract top personal to their team. When they set the right example for their team, they help foster an environment that encourages good citizenship, and motivates better performance and greater innovation.

### Study limitations

Our study has several recognized limitations. We did not incorporate specific measures of research complexity such as number of collaborating institutions and/or departments, or number of research participants per study by adding those noted as contributors in the acknowledgment sections. As such, we can only speculate whether authorship inflation occurred in original research articles independent of increased research complexity, as this inflation process is likely the reason for the increase seen in the review articles and case reports. Nevertheless, previous studies limited to specific research focuses have found evidence implying inflation as a known culprit of the growing authorship trend seen.^12^ Another obstacle we encountered in our study was designing an analysis where the specialty journals were equally “impactful.” Journal impact factor is reflective of the annual mean citations of articles published. Therefore, undertaking analysis involving only articles from “top” impact journals lends a bias to the analysis toward journals which publish more review articles and journals which are broader in scope. Despite the strict criteria for limiting journals, the citation values between specialty journals likely still vary. Journals were evaluated individually for inclusion or exclusion in our study based on content, specialty relevance, and impact factor as a surrogate for reach. Finally, article-type classifications were determined by indexed classifications in the PubMed database. However, because we evaluated articles over a 12-year period and included a diverse set of journals for analysis—article-type classification discrepancies—we have theoretically affected all specialties and journals equally, hence minimizing this potential bias in the comparative results.

## Conclusion

There has been substantial growth in the authorship bylines of contemporary medical literature, much of which cannot be fully explained by increasing research complexity alone. The increasing authorship trend is a reflection of the contemporary research landscape—one with greater complexity and collaboration and also one with more competition from lessening resources. While most are familiar with unethical publication practices such as plagiarism, falsification, and non-disclosure, less are aware of the realm of appropriate authorship. Trainees to seasoned scientists participating in medical research have likely faced or will face questions in this area. As the research environment continues to evolve with advanced tools and increased academic pressures for publication, it may be prudent to investigate how academic productivity is measured and rewarded based on contributions to quality medical literature.

## References

[1] The Economist. Why research papers have so many authors. The Economist, 24 11 2016, https://www.economist.com/news/science-and-technology/21710792-scientific-publications-are-getting-more-and-more-names-attached-them-why (accessed 25 November 2017).

[2] Pinter A. Changing authorship patterns and publishing habits in the European Journal of Pediatric Surgery: a 10-year analysis. Eur J Pediatr Surg 2015; 25(4): 353–358. 24918399 10.1055/s-0034-1376395

[3] Dang W McInnes MD Kielar AZ, et al A comprehensive analysis of authorship in radiology journals. PLoS ONE 2015; 10(9): e0139005. 26407072 10.1371/journal.pone.0139005 PMC4583466

[4] Ojerholm E Swisher-McClure S. Authorship in radiation oncology: proliferation trends over 30 years. Int J Radiat Oncol Biol Phys 2015; 93(4): 754–756. 26530742 10.1016/j.ijrobp.2015.07.2289

[5] Dubey D Sawhney A Atluru A, et al Trends in authorship based on gender and nationality in published neuroscience literature. Neurol India 2016; 64(1): 97–100. 26754999 10.4103/0028-3886.173643

[6] An JY Baiocco JA Rais-Bahrami S. Trends in the authorship of peer reviewed publications in the urology literature. Urol Pract 2017; 5(3): 233–239. 10.1016/j.urpr.2017.03.008 PMC5937928 29744377

[7] Schrock JB Kraeutler MJ McCarty EC. Trends in authorship characteristics in The American Journal of Sports Medicine, 1994 to 2014. Am J Sports Med 2016; 44(7): 1857–1860. 27159311 10.1177/0363546516639955

[8] Lehman JD Schairer WW Gu A, et al Authorship trends in 30 years of the Journal of Arthroplasty. J Arthroplasty 2017; 32(5): 1684–1687. 27998658 10.1016/j.arth.2016.11.037

[9] Valderrama-Zurian JC Aguilar-Moya R Cepeda-Benito A, et al Productivity trends and collaboration patterns: a diachronic study in the eating disorders field. PLoS ONE 2017; 12(8): e0182760. 28850569 10.1371/journal.pone.0182760 PMC5574555

[10] Khan KS Nwosu CR Khan SF, et al A controlled analysis of authorship trends over two decades. Am J Obstet Gynecol 1999; 181(2): 503–507. 10454707 10.1016/s0002-9378(99)70585-5

[11] Brunson JC Wang X Laubenbacher RC. Effects of research complexity and competition on the incidence and growth of coauthorship in biomedicine. PLoS ONE 2017; 12(3): e0173444. 28329003 10.1371/journal.pone.0173444 PMC5362051

[12] Tilak G Prasad V Jena AB. Authorship inflation in medical publications. Inquiry 2015; 52: 1–4. 10.1177/0046958015598311 PMC4943864 26228035

[13] Shaw D. Authorship inflation is unethical. EMBO Rep 2014; 15(11): 1106. 10.15252/embr.201439282 PMC4253481 25312807

[14] International Committee of Medical Journal Editors. Recommendations for the conduct, reporting, editing, and publication of scholarly work in medical journals, 12 2016, http://www.icmje.org/recommendations/25558501

[15] Association of American Medical Colleges. Table C7: full-time faculty-appointment status at U.S. medical schools for residents who completed residencies, by specialty-residents who completed training, 2008-17. Report on Residents, 2018 https://www.aamc.org/data/493938/report-on-residents-2018-c7table.html

[16] American Society of Anesthesiologists Program. Careers in medicine. Association of American Medical Colleges, 2015 https://www.aamc.org/cim/

[17] 2016 Journal Citation Reports®. Thomson Reuters, 2015, http://www.rri.res.in/htmls/library/imprints_collection/bios/files/IF%202015.pdf

[18] Number of Authors per MEDLINE®/PubMed® Citation. National Institutes of Health, 2002, https://www.nlm.nih.gov/bsd/authors1.html

[19] Green M Jones P Thomas JX Jr. Selection criteria for residency: results of a national program directors survey. Acad Med 2009; 84(3): 362–367. 19240447 10.1097/ACM.0b013e3181970c6b

[20] Hirsch JE. An index to quantify an individual’s scientific research output. Proc Natl Acad Sci U S A 2005; 102(46): 16569–16572. 16275915 10.1073/pnas.0507655102 PMC1283832

[21] Bartneck C Kokkelmans S. Detecting h-index manipulation through self-citation analysis. Scientometrics 2011; 87(1): 85–98. 21472020 10.1007/s11192-010-0306-5 PMC3043246

[22] Thelwall M Haustein S Lariviere V, et al Do Altmetrics work? Twitter and ten other social web services. PLoS ONE 2013; 8(5): e64841. 23724101 10.1371/journal.pone.0064841 PMC3665624

[23] What are Altmetrics? Altmetric, https://help.altmetric.com/support/solutions/articles/6000129311-what-is-altmetric-

[24] Guraya SY Norman RI Khoshhal KI, et al Publish or Perish mantra in the medical field: a systematic review of the reasons, consequences and remedies. Pak J Med Sci 2016; 32(6): 1562–1567. 28083065 10.12669/pjms.326.10490 PMC5216321

[25] Haileamlak A. Do authors know and follow the ICMJE recommendations? Ethiop J Health Sci 2016; 26(1): 1.

[26] Supak-Smolcic V Mlinaric A Antoncic D, et al ICMJE authorship criteria are not met in a substantial proportion of manuscripts submitted to Biochemia Medica. Biochem Med 2015; 25(3): 324–334. 10.11613/BM.2015.033 PMC4622192 26526700

[27] Rees TKM Smith S How much do healthcare professionals know about GPP authorship criteria? Curr Med Res Opin 2013; 29: S18–S19.

[28] Karani R Ognibene FP Fallar R, et al Medical students’ experiences with authorship in biomedical research: a national survey. Acad Med 2013; 88(3): 364–368. 23348080 10.1097/ACM.0b013e31827fc6ae

